# Liquid-Supported Dentures: A Soft Option—A Case Report

**DOI:** 10.1155/2013/307096

**Published:** 2013-03-11

**Authors:** Anoop Jain, Shivakumar Puranik, M. S. Jagadeesh, Puttaraj Kattimani, Savita Akki, Pawan Kumar, Vijaya laxmi'

**Affiliations:** ^1^Department of Prosthodontics, HKE'S S N Institute of Dental Sciences & Research, Gulbarga, Karnataka 585105, India; ^2^Department of Prosthodontics, Farooqia Dental College and Hospital, Mysore, Karnataka 570015, India; ^3^Department of Prosthodontics, Pandit Deendayal Upadhyay Dental College Hospital & Research Centre, Solapur, Maharastra 413255, India; ^4^Department of Prosthodontics, SB Patil Dental College & Hospital, Bidar, Karnataka 585401, India; ^5^Department of Prosthodontics, Al-Badar Dental College & Hospital, Gulbarga, Karnataka 585105, India; ^6^Department of Prosthodontics, G. Pulla Reddy Dental College, Andhra Pradesh 518002, India

## Abstract

Liquid-supported denture technique allows continued adaptation of denture to the mucosa both at resting and functional state. A complete denture prosthesis is unacceptable if it violates the foundation on which it rests. In this case, a technique for fabrication of a complete denture prosthesis that eliminates the disadvantages of tissue conditioners and soft liners (i.e., poor bond strength to acrylic, candidal colonization, etc.) and preserves the remaining tissues is described. Liquid-supported denture can be a permanent solution to some patients with problematic conditions like diabetes, xerostomia, atrophied ridge, and so forth.

## 1. Introduction

Flabby ridge is a superficial area of mobile soft tissue affecting the maxillary or mandibular alveolar ridges. It can be developed when soft tissue replaces the alveolar bone and it is a common finding, particularly in long term denture wearers [1]. These soft tissue changes and bone resorption occurs because of muscle dynamics or tissue irritation, which ultimately affects the residual ridge dimensions [2]. Thus, complete denture seldom remain in close adaptation to the adjacent mucosa. However, it also has to support the teeth during function and thus should be rigid. An ideal denture base should be flexible, as it has to continuously adapt to the mucosa. However, it also has to be rigid so as to support the teeth during function. These properties cannot be combined in one material, but are possible by using combination of materials [3]. 

Several techniques have been tried on the tissue surface of the dentures. In 1961, Chase introduced the use of elastic impression material to relieve traumatized tissue. But this can be only a temporary provision. Moreover, it might easily derive candidal growth. Since then a variety of tissue conditioning materials has been introduced. Another group of materials called soft liners has been used to relieve “denture sore mouth” problems. But soft liners are also only temporary provisions because due to loss of plasticizer over a period of time, they lose their plastic properties. In a flabby ridge condition, an ideal denture should be able to withstand masticatory forces and have flexible tissue surface to reduce stress concentration and trauma on the underlying tissues [4]. A Liquid-supported denture can hence be a solution for this problem. This paper describes the fabrication of a mandibular complete denture with its base covered with a preshaped, close fitting, and flexible sheet containing a thin film of viscous liquid, which fulfills the aforementioned requirements.

## 2. Case Report

**Figure 1:**
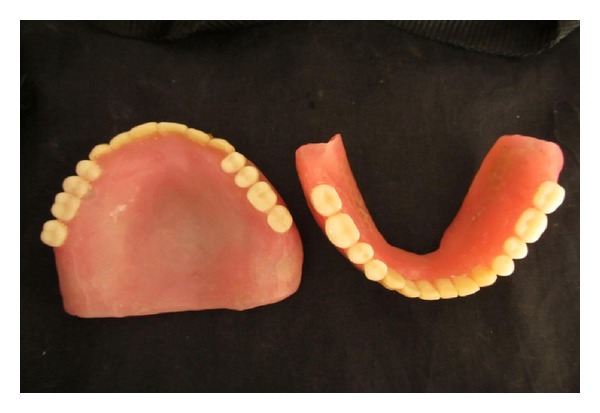
Patient's old denture.

**Figure 2:**
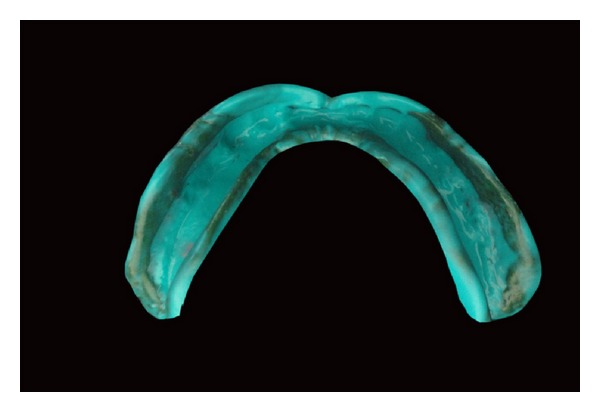
Final impression made with elastomeric impression material.

A 56-year-old male patient reported to HKE'S S N Institute of Dental sciences & Research, Gulbarga, Karnataka, India, for replacement of missing teeth. The patient had a history of wearing maxillary and mandibular complete dentures since the past 6 years (Figure 1). His chief complaint was the poor fit of the denture, and it felt loose while eating. Patient had a history of diabetes and hypertension since last 9 years. The patient was wearing complete dentures even at the night. He was also using denture adhesive. By intraoral examination, a completely edentulous mandibular arch with flabby tissue existing in the mandibular anterior region was observed. It was decided to give a conventional maxillary complete denture opposing a Liquid-supported mandibular complete denture because of flabby soft tissues in anterior mandibular region. Primary impressions were made with alginate (Prime Dental products pvt. Ltd., Mumbai, India). Full complete double spacer was applied in the mandibular anterior region because of flabby soft tissues in the anterior region. Border molding was performed by using low fusing impression compound (DPI pinnacle tracing sticks, Dental products of India), and final impression was made with light body additional silicone impression material (Aquasil, Dentsply/caulk) (Figure 2). Denture base of 3 mm thickness was fabricated for mandibular denture base and 2mm for maxillary 3 denture base. Jaw relations record and trial of waxed up denture was done by conventional method. The mandibular denture design was modified to make a Liquid-supported denture. Maxillary complete denture was acrylised using conventional procedure.

Steps in fabricating a Liquid-supported denture.

**Figure 3:**
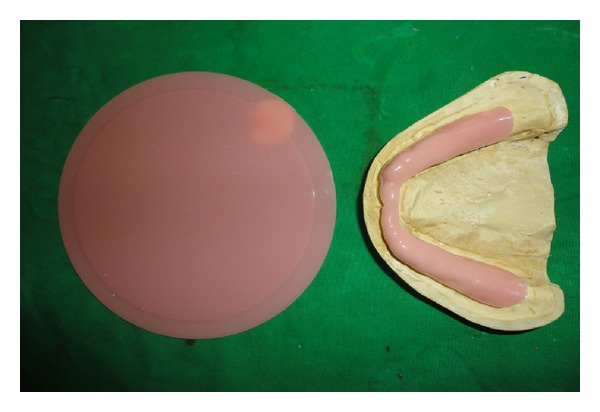
Vacuum heat-pressed polyethylene sheet (1.5 mm thick).

**Figure 4:**
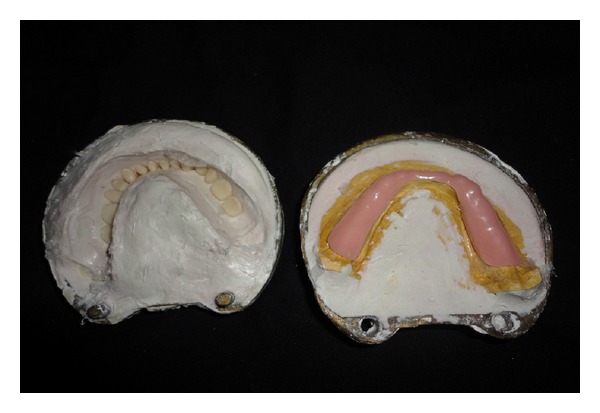
Vacuum heat-pressed polyethylene sheet (1.5 mm thick) incorporated at the time of packing.

**Figure 5:**
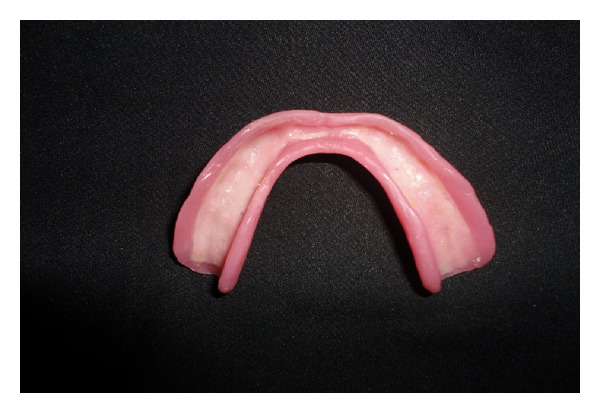
Polished denture with 1.5 mm thick temporary polyethylene sheet.

**Figure 6:**
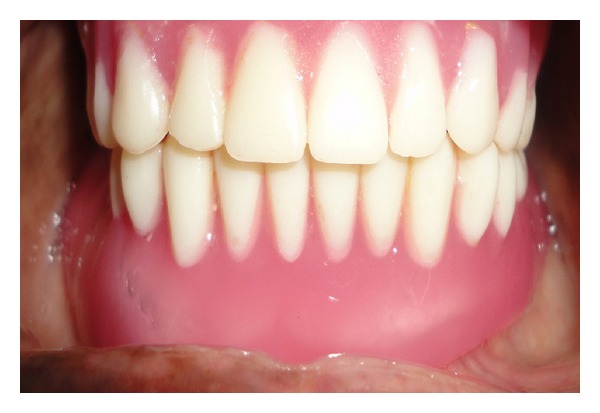
Intraoral view of maxillary and mandibular complete dentures.

**Figure 7:**
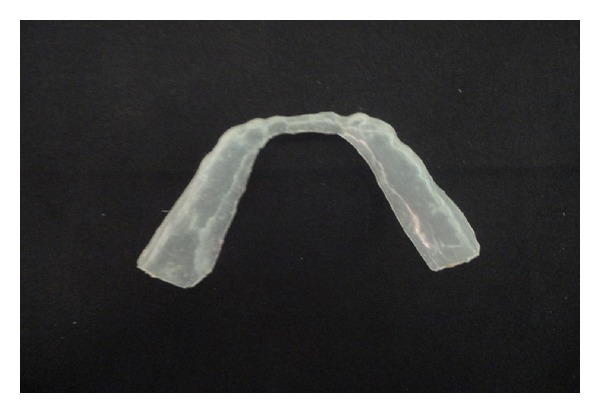
Vacuum heat-pressed 0.5 thick final polyethylene sheet.

**Figure 8:**
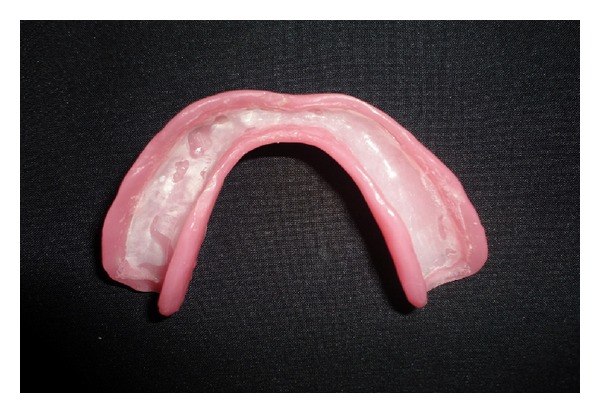
Liquid-supported mandibular denture with 0.5 mm thick final polyethylene sheet filled with glycerine (tissue surface).

**Figure 9:**
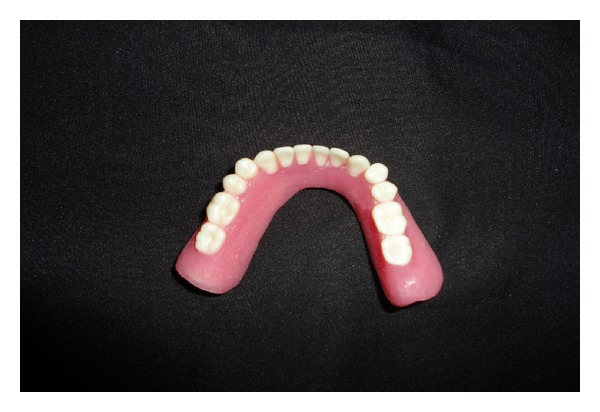
Final mandibular denture (Polished surface).

1. Vacuum heat-pressed polyethylene sheet (Biostar vacuum forming machine, Scheu-dental, Germany) of 1.5 mm thickness was adapted on the mandibular master cast. The sheet acted as a temporary spacer, and it was made 2 mm short of the sulcus (Figure 3).
2. After dewaxing, 1.5mm temporary polyethylene sheet was adapted on the mandibular cast, and vaseline was applied over it so that this temporary sheet can be retrieved easily (Figure 4). Now, the denture was acrylised using heat cure resin along with the sheet. The mandibular denture with this 1.5 mm thick temporary polyethylene sheet was then finished and polished in conventional manner (Figure 5).
3. The mandibular denture was inserted in to the patient's mouth to check for retention, support, stability, and border extension. The patient was asked to wear the denture for two weeks to get adjusted to it (Figure 6).
4. After two weeks, the patient was recalled to convert the mandibular denture into a Liquid-supported one. Temporary polyethylene 1.5 mm thick spacer sheet was removed from the mandibular denture.
5. An additional silicone putty impression was made of the tissue surface of the denture, and the cast was made of it. This was done to record the exact junction of the sheet to the denture. The impression was poured with dental stone, which will form negative replica of the ridge. Now, again the cast was poured upon the negative replica to produce positive replica of the ridge. On this cast, a 0.5 mm thick final polyethylene sheet was vacuum heat pressed which was used in place of 1.5 mm thick sheet creating a 1 mm space between tissue surface of the denture and permanent polyethylene sheet (Figure 7).
6. The polyethylene sheet was cut using the putty index as guide. The borders of the 0.5 mm thick sheet were placed in the crevice formed due to removal of 1.5 mm thick sheet. Cyanoacrylate adhesive and autopolymerizing acrylic resin were used to seal the borders and prevent escape of liquid (Figure 8).
7. The space created due to the replacement of a 1.5 mm thick sheet with a 0.5 mm thick sheet was filled with viscous liquid, that is, glycerine. This was done by making two holes drilled on the buccal flange in the molar area of the denture by round bur and injecting viscous liquid, that is, glycerine through these holes, and one hole was sealed with autopolymerizing cure acrylic resin.
8. The occlusal vertical dimension was adjusted by fitting the denture in the patient's mouth, and the other hole was sealed using the autopolymerizing cure acrylic resin. Then, the mandibular Liquid-supported denture was delivered (Figure 9).
9. Denture care instructions were given to the patient. The patient was told to clean the tissue surface using cotton. The patient was recalled for followup.

## 3. Discussion

**Figure 10:**
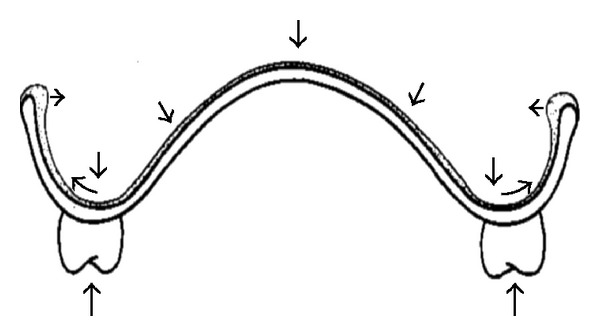
Unidirectional loading of denture resulting in multidirectional distribution of hydrodynamic pressure throughout fluid and clasping pressure at border of denture.

In this case where the presence of flabby tissue in mandibular arch (anterior portion) was treated by a modified design, that is, Liquid-supported denture. The principle of this design was that a Liquid-supported denture is flexible and continuously adapts itself to the mucosa. However, it is also rigid enough to support the teeth during actual use. Thus, the denture base is covered with a close-fitting flexible foil to keep a thin film of liquid in its place. This design will act as a continuous reliner for the denture and thus has an advantage over the existing denture designs. When no forces are applied, the foil remains in the resting position and act as a soft liner, and when the dentures are in use, vertically directed loads are distributed in all directions by the liquid resulting in optimal stress distribution (Figure 10). Thus, Liquid-supported denture provides benefits of both tissue conditioners and soft liners. This helps in long-term the preservation of bone and soft tissues. Apart from the combined benefits of tissue conditioners and soft liners, the load from biting forces and even bruxism will be distributed over a larger surface [5].

*The Precautions are as follows (see [6]):*

- thickness of denture base should be at least 3 mm;
- The seal should be perfect and should be checked for microleakage;
- denture care instructions should be given to the patient;
- in case the liquid leaks out, the patient should inform the dentist, and the denture should be refilled;
- repair is possible if the sheet gets ruptured and can be replaced over preserved stone cast.

For a liquid cushion, glycerine was used, which is clear, colorless, and odourless with a good pharmaceutical action. To prevent the liquid from leakage, a dense foil must be used [7]. The problem faced in fabrication of complete denture is the difficulty in a achieving complete seal at the junction of polyethylene sheet and denture base. The main drawback of liquid-supported denture is the relining procedure, which is not possible with this liquid supported denture [8].

## 4. Conclusion

Days and nights change, men, tissues, so do our treatments. Ultimately, Devan's dictum holds true “Our objective should be perpetual preservation of what remains, rather than meticulous reconstruction of what is lost.” The seeds of success or failure of the prosthesis lies in the hands of the dentist. Liquid-supported denture by acting as a continuous reline provides solution to some problematic prosthodontic situations like patients with bruxism or clenching habits, in xerostomia patients, or those patients with atrophic ridge, superficial mental nerve, and so forth. Liquid-supported denture with its shock absorbing effect thus fulfills a valuable role in prosthetic dentistry.
